# Suicide deaths and substance use in the Galician provinces between 2006 and 2020

**DOI:** 10.3389/fpsyt.2023.1242069

**Published:** 2023-08-14

**Authors:** Gerardo Flórez, Ashkan Espandian, Noelia Llorens, Teresa Seoane-Pillado, Pilar Saiz

**Affiliations:** ^1^Addictive Treatment Unit, Ourense University Hospital, Ourense, Spain; ^2^Centre for Biomedical Research in the Mental Health Network (CIBERSAM), Madrid, Spain; ^3^Psychiatry Service, Bierzo Hospital, Ponferrada, Spain; ^4^Spanish Observatory of Drugs and Addictions, Government Delegation for the National Plan on Drugs, Spanish Ministry of Health, Madrid, Spain; ^5^Area of Preventive Medicine and Public Health, Department of Health Sciences, University of A Coruña – INIBIC, A Coruña, Spain; ^6^Department of Psychiatry, University of Oviedo, Oviedo, Spain; ^7^Health Research Institute of the Principality of Asturias (ISPA), Asturias, Spain

**Keywords:** alcohol, completed suicide, illegal substances, Joinpoint regression, preventive strategies

## Abstract

**Background:**

Suicide is a serious public health problem that affects our entire country, including the Galician provinces. The aim of this research was to study the variation in completed suicide rates, between 2006 and 2020, in the different Galician provinces and their relationship with the consumption of addictive substances.

**Methods:**

Completed suicide data from the Spanish Office for National Statistics and the Institute of Legal Medicine of Galicia were analyzed with a Joinpoint regression model to determine time trends. The relationship between the variation in completed suicide rates with sociodemographic variables obtained from the Spanish Office for National Statistics and variables related to the consumption of substances obtained from the survey on alcohol and other drugs in Spain (EDADES) of the Government Delegation for the National Plan on Drugs was also analyzed.

**Results:**

The Joinpoint regression model did not reveal any point of significant change in the period studied for any Galician province. The following variables correlated positively with the variation in completed suicide rates in the Galician provinces: masculinity ratio, average age, daily alcohol consumption and daily illegal substance consumption.

**Conclusion:**

Applying preventive strategies on the daily consumption of alcohol and illegal substances would help reduce the rates of completed suicide in the Galician provinces.

## Introduction

Suicide and substance abuse represent two serious problems in the health system and in society. Both problems extend globally.

According to the World Health Organization (WHO), the number of deaths by suicide stands at approximately 700,000 people per year, although it is estimated to be higher due to the underreporting that occurs in the different registries (1).

The global rate is estimated at 9.4 suicides per 100,000 population with a higher percentage in the male sex and with increasing age. It is considered the leading cause of death within the population aged 15–34 years old (2).

In Spain, according to data from the (3), suicide has been the leading external cause of mortality since 2008 and exceeds deaths because of traffic accidents (4). In Spanish autonomous communities, the high suicide rates in Asturias and Galicia stand out with a percentage of 12.85 and 12.50 per 100,000 inhabitants, respectively, (3).

Suicide is the leading cause of death in people with substance use disorder (5). It is estimated that the risk of death by suicide compared to the general population increases 10-fold and 14-fold for alcohol use disorder and addiction to other substances, respectively (6).

According to the Spanish Observatory on Drugs and Addictions^1^ these were the trends in 2019/2020 of use in Spain of the main psychoactive substances of abuse: Alcohol continues to be the most commonly consumed psychoactive substance. 77.2% have consumed alcohol in the last 12 months, 63.0% in the last 30 days and 8.8% daily in the last 30 days. 39.4% of the population aged 15–64 has used tobacco in the last year, 36.8% in the last month and 32.3% on a daily basis. Compared to 2017, the figure has decreased from 34.0 to 32.3%, returning to the figures recorded in 2005, after which there was a decrease over the years until 2017. In 2019/2020, 10.5% of the population aged 15–64 has tried e-cigarettes and, within this age group, 48.9% has used e-cigarettes with nicotine, whereas 24.3% smoked both nicotine and nicotine-free cartridges. Regarding prescribed and non-prescribed hypnosedatives, an estimated 645,000 people started using them in 2019/2020, with the highest uptake among women aged 35–64. 15.2% of the Spanish population aged 15–64 years admits having used opioid analgesics with or without a prescription at some point in their lives. 37.5% of the population aged 15–64 has ever used cannabis, 10.5% in the last year, 8.0% in the last month (for the time periods of last year and last month prevalence has remained stable since 2001) and 2.9% daily in the last month. 10.9% of the population aged 15–64 has ever used powder cocaine, 2.5% in the last year and 1.1% in the last 30 days. In 2019/2020, 0.7% of the population aged 15–64 years has used heroin at some time, 0.1% in the last year and 0.0% in the last month. In 2019/2020, 5.5% of the population aged 15–64 has ever used hallucinogens, 4.3% amphetamines and 5.0% ecstasy.

Alcohol plays a relevant role, not only as a risk factor, but also as a precipitating factor, due to the disinhibition and executive dysfunction produced by alcohol intoxication (7). The increased risk is estimated to be so significant that a population’s overall alcohol consumption is associated with the prevalence of suicidal risk (8, 9).

In terms of suicide prevention strategies, it is especially relevant to note that to date, of all the strategies that have been used, very few enjoy any degree of sufficient evidence to be able to ensure that they are able to reduce the incidence of suicide (10, 11). Work continues to identify a cornerstone intervention (12, 13).

The main aim of this study is to determine the relationship between the variation in completed suicide rates in the Galician provinces between 2006 and 2020 and active substance use variables in these provinces. A secondary objective would be to perform the same evaluation, but for other variables indirectly related to substance use.

The working hypothesis is that active substance use has a significant influence on the variation in completed suicide rates in the Galician provinces between 2006 and 2020. If this hypothesis is verified, the importance of globally reducing the consumption of psychoactive substances as a preventive measure against suicide would be confirmed in the study population.

## Materials and methods

### Data

The incidence rates of suicide deaths for the four Galician provinces between 2006 and 2020 were obtained from two databases (Tables 1, 2). First, from IMELGA (Institute of Legal Medicine of Galicia)^2^ and second from the Spanish Office for National Statistics (INE).^3^ Given the disparity found between the two sources, they were compared separately.

**Table 1:** Prevalence of suicide deaths in the 4 Galician provinces from 2006 to 2020 according to the IMELGA.

	La Coruña (1)	Lugo (2)	Orense (3)	Pontevedra (4)	Statistical significance
2006	14.61	8.69	6.73	9.01	1 vs. 3 (*p* < 0.001) 1 vs. 4 (*p* < 0.001)
2007	9.18	14.92	12.38	8.96	2 vs. 4 (*p* = 0.003)
2008	9.21	11.25	11.81	8.70	
2009	13.26	15.20	13.79	11.14	2 vs. 1 (*p* = 0.001)
2010	12.82	20.25	7.63	6.23	1 vs. 3 (*p* = 0.012) 1 vs. 4 (*p* < 0.001) 2 vs. 3 (*p* < 0.001) 2 vs. 4 (*p* < 0.001)
2011	12.46	14.22	14.50	8.40	1 vs. 4 (*p* = 0.004) 2 vs. 4 (*p* = 0.003)
2012	15.47	15.76	12.61	9.18	1 vs. 4 (*p* < 0.001) 2 vs. 4 (*p* = 0.001) 3 vs. 4 (*p* = 0.002)
2013	12.30	15.02	13.87	11.20	
2014	15.97	17.50	9.04	12.82	1 vs. 3 (*p* = 0.003) 2 vs. 3 (*p* = 0.002) 2 vs. 4 (*p* = 0.0048)
2015	13.97	17.08	9.73	8.65	1 vs. 4 (*p* = 0.001) 2 vs. 3 (*p* = 0.01) 2 vs. 4 (*p* < 0.001)
2016	15.94	9.80	11.11	10.37	1 vs. 3 (*p* = 0.05) 1 vs. 4 (*p* = 0.001)
2017	13.30	13.18	14.75	9.01	2 vs. 1 (*p* = 0.03) 1 vs. 4 (*p* = 0.004) 2 vs. 4 (*p* = 0.004) 3 vs. 4 (*p* = 0.007)
2018	11.53	14.18	12.93	7.64	1 vs. 4 (*p* = 0.005) 2 vs. 4 (*p* = 0.001) 3 vs. 4 (*p* = 0.007)
2019	12.77	17.59	11.05	8.80	2 vs. 1 (*p* = 0.045) 1 vs. 4 (*p* < 0.001) 2 vs. 3 (*p* = 0.03) 2 vs. 4 (*p* < 0.001)
2020	12.03	13.11	10.10	10.36	

Vs, versus.

**Table 2:** Prevalence of suicide deaths in the 4 Galician provinces from 2006 to 2020 according to INE.

	La Coruña (1)	Lugo (2)	Orense (3)	Pontevedra (4)	Statistical significance
2006	12.31	14.58	7.97	8.69	1 vs. 3 (*p* = 0.037) 1 vs. 4 (*p* = 0.012) 2 vs. 3 (*p* = 0.01) 2 vs. 4 (*p* = 0.003)
2007	11.65	15.20	12.46	8.01	1 vs. 4 (*p* = 0.009) 2 vs. 4 (*p* < 0.001) 3 vs. 4 (*p* = 0.021)
2008	11.41	12.37	11.00	8.91	
2009	13.79	12.38	10.72	10.62	1 vs. 4 (*p* = 0.039)
2010	10.90	18.10	6.56	6.64	2 vs. 1 (*p* = 0.001) 1 vs. 3 (*p* = 0.026) 1 vs. 4 (*p* = 0.001) 2 vs. 3 (*p* < 0.001) 2 vs. 4 (*p* < 0.001)
2011	11.68	13.37	12.90	8.51	1 vs. 4 (*p* = 0.023) 2 vs. 4 (*p* = 0.013) 3 vs. 4 (*p* = 0.026)
2012	13.11	16.05	11.80	9.07	1 vs. 4 (*p* = 0.006) 2 vs. 4 (*p* = 0.001)
2013	11.77	15.02	11.63	11.41	
2014	15.09	17.79	8.99	11.56	1 vs. 3 (*p* = 0.009) 1 vs. 4 (*p* = 0.029) 2 vs. 3 (*p* = 0.002) 2 vs. 4 (*p* = 0.006)
2015	13.48	14.73	8.79	9.28	1 vs. 3 (*p* = 0.036) 1 vs. 4 (*p* = 0.015) 2 vs. 3 (*p* = 0.027) 2 vs. 4 (*p* = 0.008)
2016	14.42	10.69	10.16	11.43	
2017	12.40	17.38	11.87	9.44	2 vs. 1 (*p* = 0.03) 1 vs. 4 (*p* = 0.044) 2 vs. 4 (*p* < 0.001)
2018	11.16	12.67	10.66	7.85	1 vs. 4 (*p* = 0.016) 2 vs. 4 (*p* = 0.002)
2019	11.61	16.08	9.42	8.48	2 vs. 1 (*p* = 0.045) 1 vs. 4 (*p* = 0.026) 2 vs. 3 (*p* = 0.019) 2 vs. 4 (*p* < 0.001)
2020	11.23	13.72	10.10	10.89	

Vs, versus.

The following data were also obtained by means of the INE database for the four Galician provinces: unemployment rates, masculinity ratio (number of men for every 100 women), average life expectancy and mean age (Table 3).

**Table 3:** Rates of sociodemographic variables obtained from the INE with ANOVA comparisons between provinces.

	2006	2007	2008	2009	2010	2011	2012	2013	2014	2015	2016	2017	2018	2019	2020
U															
La Coruña	8.12	8.22	8.91	11.47	13.97	15.82	18.38	20.18	19.95	17.40	15.41	14.16	12.23	10.20	11.23
Lugo	6.56	5.76	6.28	9.40	10.78	11.90	16.11	18.96	18.94	15.95	14.78	12.66	9.18	8.86	8.78
Orense	8.98	5.77	6.25	10.41	16.51	17.77	20.96	24.06	21.34	20.34	19.54	16.47	13.75	12.93	11.72
Pontevedra	9.04	8.01	9.88	15.25	18.19	20.70	24.50	24.74	24.70	22.46	19.38	18.31	16.01	14.29	14.13
ANOVA	There are significant differences between Lugo and Pontevedra (*p* = 0.037)														
MR															
La Coruña	92.21	92.31	92.52	92.65	92.71	92.74	92.79	92.81	92.75	92.72	92.62	92.51	92.44	92.37	92.35
Lugo	94.07	94.25	94.51	94.86	94.85	94.76	94.80	94.59	94.47	94.47	94.48	94.49	94.47	94.35	94.33
Orense	92.35	92.40	92.68	92.89	93.04	93.31	93.43	93.49	93.39	93.29	93.18	93.23	93.15	92.97	92.93
Pontevedra	93.25	93.41	93.63	93.86	94.10	94.30	94.48	94.48	94.45	94.41	94.42	94.39	94.23	94.17	94.08
ANOVA	There are significant differences between La Coruña and Pontevedra (*p* < 0.0001), between La Coruña and Lugo (*p* < 0.0001), between Orense and Lugo (*p* < 0.0001) and between Orense and Pontevedra (*p* = 0.022)														
MA															
La Coruña	43.69	43.94	44.14	44.34	44.56	44.79	45.05	45.37	45.63	45.94	46.21	46.46	46.71	46.92	47.19
Lugo	47.47	47.69	47.80	47.94	48.11	48.32	48.53	48.70	48.93	49.18	49.36	49.54	49.69	49.86	50.10
Orense	47.78	48.01	48.21	48.33	48.51	48.73	48.96	49.15	49.43	49.73	49.98	50.22	50.45	50.65	50.86
Pontevedra	41.84	42.08	42.34	42.56	42.83	43.12	43.40	43.70	44.02	44.37	44.70	45.02	45.29	45.60	45.91
ANOVA	There are significant differences between La Coruña and Lugo (*p* = 0.002), between La Coruña and Ourense (*p* < 0.0001), between Pontevedra and Lugo (*p* < 0.0001) and between Pontevedra and Ourense (*p* < 0.0001)														
ALE															
La Coruña	80.65	80.54	81.07	81.16	81.69	81.95	82.21	82.34	82.62	82.33	82.53	82.93	82.77	83.36	83.44
Lugo	80.81	81.15	81.51	81.24	82.06	82.28	81.91	82.66	82.66	82.38	82.56	82.98	82.89	83.68	83.06
Orense	81.17	81.46	81.43	82.24	82.22	81.90	82.39	82.63	83.66	83.20	83.05	83.77	83.29	83.57	83.05
Pontevedra	80.72	80.90	81.38	81.52	81.97	82.18	82.20	82.79	83.07	82.78	82.66	83.02	83.44	83.58	83.39
ANOVA	There are no significant differences														

U, Unemployment; MR, Masculinity ratio; MA, Mean Age; ALE, Average Life Expectancy.

The data on substance use for the same period for the four Galician provinces come from the survey on alcohol and other drugs in Spain (EDADES)^4^, which the Government Delegation for the National Plan on Drugs has performed biannually since 1995 (Table 4). The following variables were included: alcohol use in the last 30 days, daily alcohol use, binge drinking in the last 12 months, Positive Alcohol Use Disorder Test (AUDIT) for risky alcohol use, Daily benzodiazepine (BZD) use, Daily use of illegal substances, Mortality associated with substance use in the Specific Mortality Register (SMR) and Mortality associated with substance use in the General Mortality Register (GMR).

**Table 4:** Rates of variables associated with substance use obtained from the PNSD.

	2006	2007	2008	2009	2010	2011	2012	2013	2014	2015	2016	2017	2018	2019	2020
A-30 days															
La Coruña						56.7		64.6		65.5			63.2		60.3
Lugo						68.1		68.6		63.7			66.4		68.5
Orense						66.5		64.8		69.5			65.9		73.5
Pontevedra						64.8		70.0		64.6			69.5		64.6
ANOVA	There are no significant differences														
A-daily															
La Coruña						13.8		12.5		13.2			6.9		7.7
Lugo						27.2		15.3		16.4			7.9		12.5
Orense						23.9		19.0		19.9			14.4		21.5
Pontevedra						20.9		13.1		10.3			6.3		9.6
ANOVA	There are no significant differences														
Binge															
La Coruña						17.2		24.0		25.1			15.5		20.2
Lugo						20.3		21.3		18.8			29.1		20.7
Orense						25.6		24.4		24.9			26.0		30.0
Pontevedra						19.1		18.6		21.0			18.2		10.89
ANOVA	There are significant differences between Pontevedra and Orense (*p* = 0.017)														
AUDIT+															
La Coruña				6.0				11.1					4.2		7.2
Lugo				4.1				8.7					8.6		5.7
Orense				3.2				7.7					9.0		12.8
Pontevedra				4.4				7.0					7.6		3.6
ANOVA	There are no significant differences														
BZD-daily															
La Coruña						4.6		7.9		9.8			8.8		8.7
Lugo						8.5		6.5		10.4			4.7		7.2
Orense						6.8		10.6		7.2			5.3		13.4
Pontevedra						9.0		9.7		9.0			4.6		8.2
ANOVA	There are no significant differences														
IS- daily															
La Coruña						2.5		5.7		10.1			8.1		6.2
Lugo						4.5		5.3		3.3			9.2		5.5
Orense						5.1		5.9		7.4			6.9		13.3
Pontevedra						6.6		7.4		7.1			10.9		6.8
ANOVA	There are no significant differences														
Mort-SMR															
La Coruña	1.31	2.64	2.45	2.35	2.96				1.94	2.30	1.87	1.60	2.85	3.84	3.92
Lugo	0.56	2.25	1.96	1.40	3.37				2.91	1.76	2.37	2.09	2.71	2.42	3.65
Orense	0.29	0.89	0.98	1.48	1.19				0.0	0.62	1.58	1.28	0.32	1.95	1.30
Pontevedra	2.01	2.42	2.93	1.97	1.87				1.89	2.63	2.01	1.90	1.59	2.12	3.17
ANOVA	There are significant differences between Pontevedra and Ourense (*p* = 0.004), between Pontevedra and Lugo (*p* = 0.01) and between Pontevedra and La Coruña (*p* < 0.0001)														
Mort-GMR															
La Coruña	1.06	2.91	1.75	0.87	0.61				0.97	0.62	0.80	0.89	1.16	1.69	3.83
Lugo	0.56	2.25	0.28	0.84	0.56				2.62	0.0	0.29	0.29	0.0	0.30	2.43
Orense	1.77	1.18	1.48	1.19	0.59				0.93	0.62	0.0	0.0	0.64	0.97	0.97
Pontevedra	1.59	3.37	2.09	1.04	0.93				2.10	0.84	2.01	1.16	0.74	1.27	3.27
ANOVA	There are significant differences between Lugo and Pontevedra (*p* = 0.024)														

A-30 días, Alcohol use in the last 30 days; A-daily, Daily alcohol use; Binge, Binge drinking in the last 12 months; AUDIT+, Positive Alcohol Use Disorder Test (AUDIT) for risky alcohol use; BZD-Daily, Daily benzodiazepine (BZD) use; IS-daily, Daily use of illegal substances; Mort-SMR, Mortality associated with substance use in the Specific Mortality Register (SMR); Mort-GMR, Mortality associated with substance use in the General Mortality Register (GMR).

Finally, another variable to consider is the Intensive Intervention Programme for suicidal behavior. An outpatient programme launched in the province of Ourense since 2009, with the aim of reducing mortality from suicide and suicide attempts in the province (14). During the study period, there was no similar programme in the other Galician provinces.

### Ethics aspects

The researchers complied with all the contents set out in the current legislation on clinical research established in the Declaration of Helsinki, Council of Europe Convention on Human Rights and Biomedicine and in the UNESCO Universal Declaration on Human Rights. They complied with the requirements established in Spanish legislation in the field of medical research, the protection of personal data and bioethics and all other requirements set out by Spanish legislation on this topic. Written informed consent for participation was not required for this study in accordance with the national legislation and the institutional requirements. The databases used are 100% anonymous, there is no ethical problem in their management, in fact they are governmental and accessible to all citizens. The current research contains no human or animal studies.

### Statistical analysis

The temporal trend of completed suicide incidence rates in each Galician province was evaluated using a Joinpoint regression model. This statistical modeling technique is ideal to analyze the appearance of temporal changes and trends over lengthy periods of time and has previously been used successfully in the field of addictions (15, 16). Changes in the time trend are called Joinpoints or inflection points. Statistical criteria determine the final number of Joinpoints. A Poisson distribution model was used in the estimation. This regression model also enables calculation of the percentage annual change (PAC). Joinpoint analysis also facilitates two-by-two comparisons. It performs the test on the slopes by testing whether the two functions are parallel (parallelism test).

A locally weighted smoothing (LOESS) regression model was used to compare the prevalence rates of completed suicide with the consumption variables obtained from the EDADES survey database and the complementary variables from the INE. These data are used as covariates to model the incidence rates of completed suicide. The model’s *p*-value indicates whether the LOESS is statistically significant. This technique makes it possible to see the oscillations of the suicide incidence rates as a function of the covariates.

The completed suicide rates and the rest of the variables were also compared between provinces applying ANOVA comparisons (non-parametric test for sample size) with *post-hoc* analysis of two-by-two comparison.

The criterion of statistical significance in all tests was *p* ≤ 0.05, established as the maximum acceptable value for the probability of making a type 1 error.

## Results

Table 1 shows the prevalence of completed suicide in the four Galician provinces according to IMELGA data and the significant differences between them. Table 2 shows the prevalence of completed suicide in the four Galician provinces according to the INE data and the significant differences between them. It should be noted that the ANOVA test also obtained the following statistically significant results when comparing completed suicide rates between provinces, according to IMELGA. There are statistically significant differences between La Coruña and Pontevedra (*p* = 0.002) and between Lugo and Pontevedra (*p* < 0.0001). According to the INE, there are statistically significant differences between La Coruña and Pontevedra (*p* = 0.001), between Lugo and Pontevedra (*p* < 0.0001) and between Lugo and Orense (*p* < 0.0001). Table 3 shows the rates of sociodemographic variables obtained from the INE and the ANOVA significant results between provinces. Table 4 shows the rates of substance use variables obtained from the PNSD and the ANOVA significant results between provinces.

**Figure 1:**
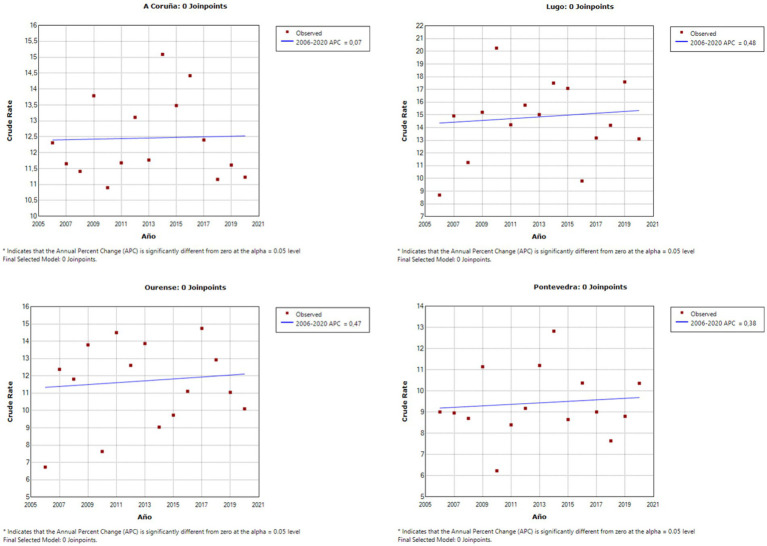
Joinpoint regression models in each Galician province for suicide rates according to the IMELGA.

**Figure 2:**
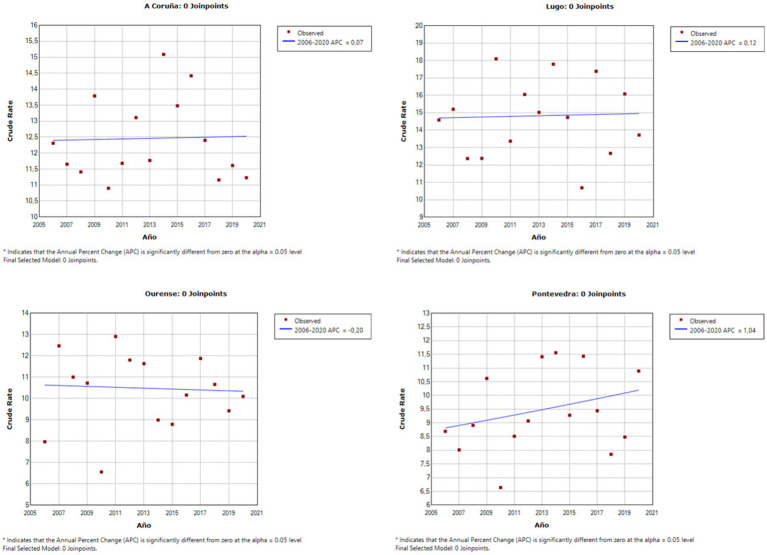
Joinpoint regression models in each Galician province for completed suicide rates according to the INE.

**Figure 3:**
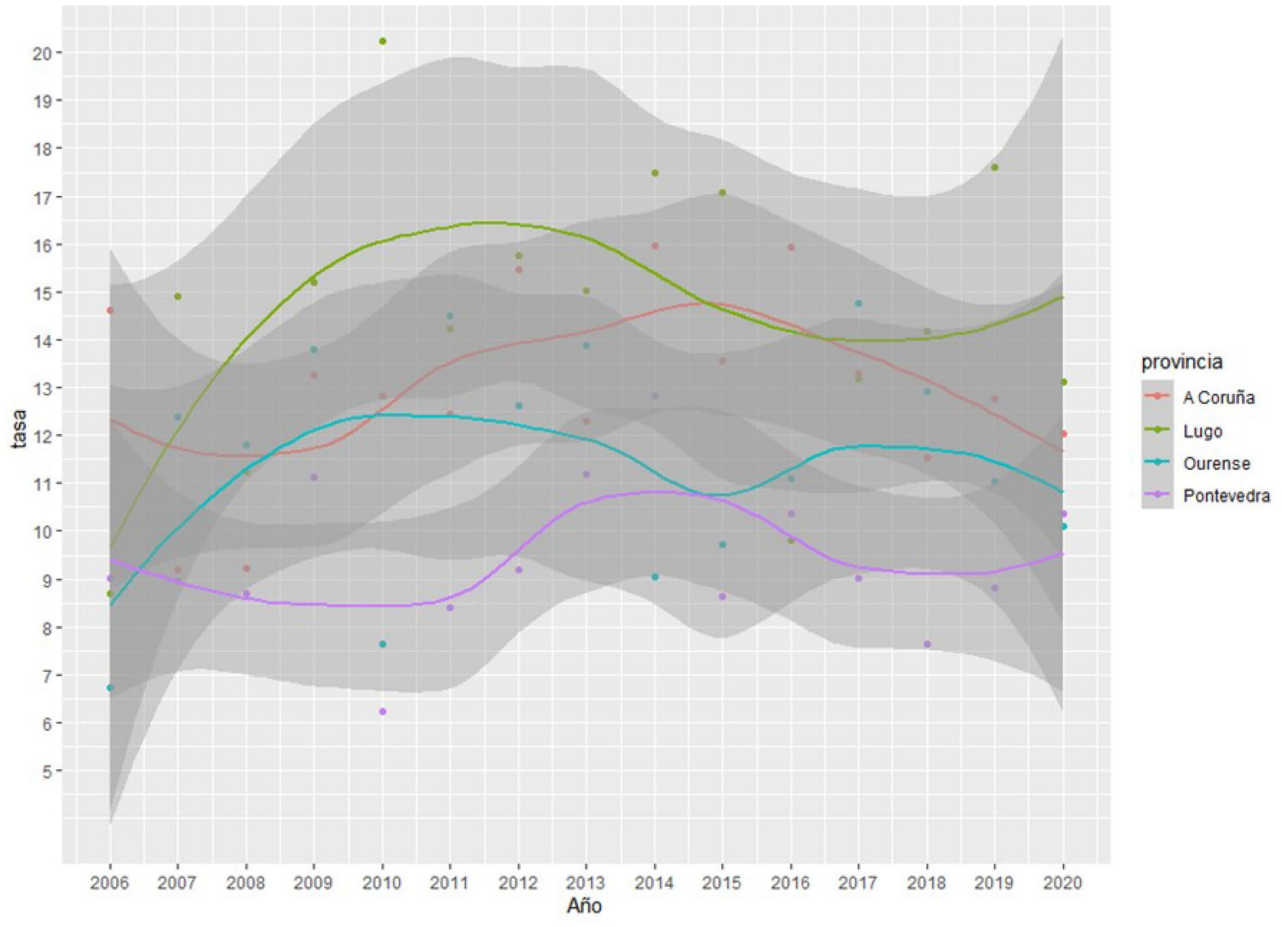
Evolution of completed suicide rates in the four Galician provinces according to IMELGA during the study period.

**Figure 4:**
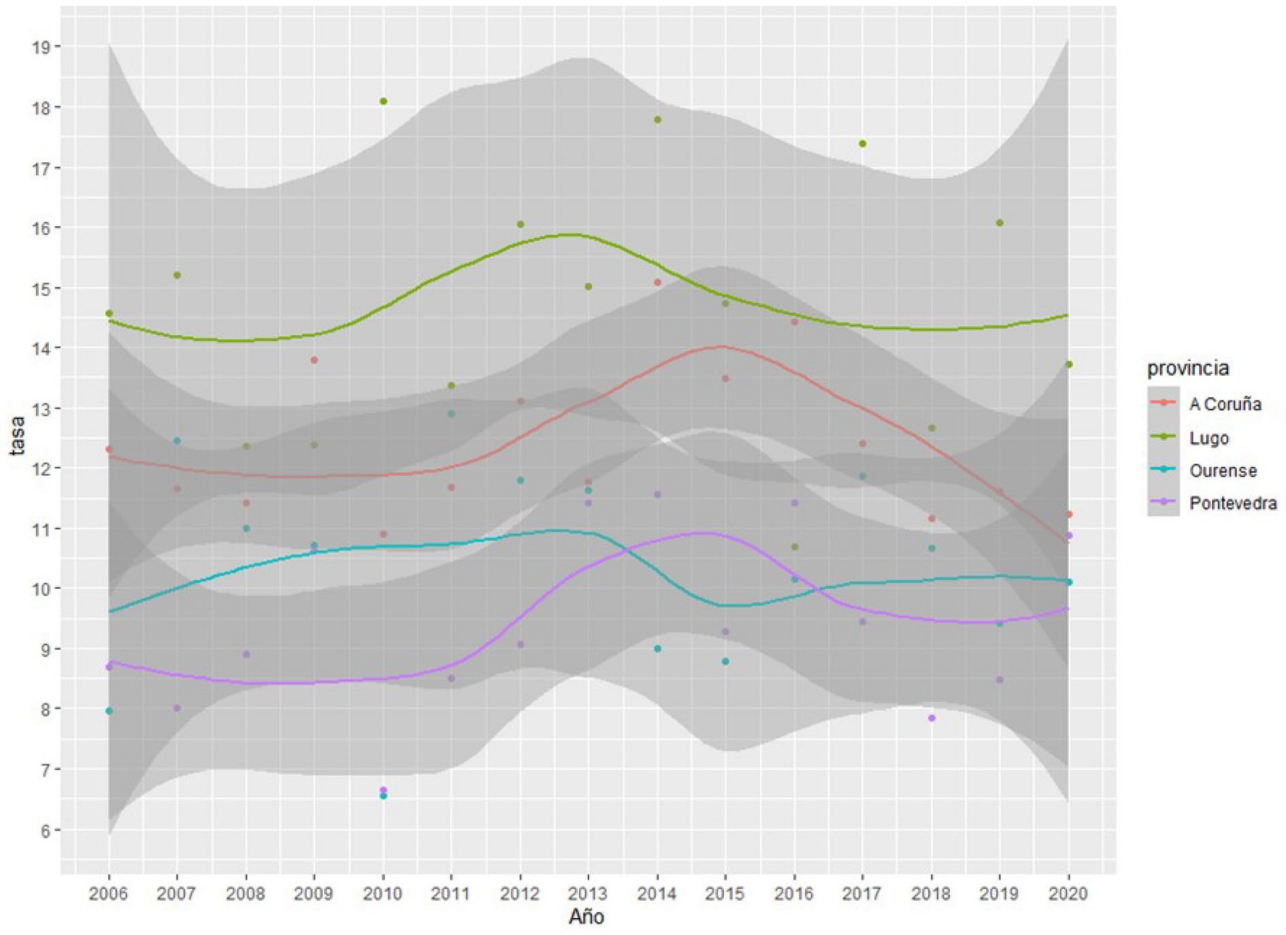
Evolution of completed suicide rates in the four Galician provinces according to INE during the study period.

As can be observed in Figures 1, 2, the Joinpoint regression model did not lead to identification of any significant change points for the completed suicide rates in any Galician province in both databases. Figures 3, 4 show the variation in completed suicide rates in each Galician province throughout the study period for each database. In the two-to-two Joinpoint comparison, performing the contrast on the best estimated model, results are obtained that indicate that the option of parallelism cannot be ruled out. The LOESS regression models yield the following significant results for the IMELGA data: mean age: 5.53 (*p* = 0.005), masculinity ratio: 3.99 (*p* = 0.005), daily alcohol consumption: 8.98 (*p* < 0.001) and daily consumption of illegal substances: 4.89 (*p* = 0.006). And for the INE data, average age: 4.8 (*p* = 0.008), masculinity ratio: 4.4 (*p* = 0.004), daily alcohol consumption: 5.34 (*p* = 0.007) and daily consumption of illegal substances: 5.47 (*p* = 0.005). Figures 3, 4 show the variation in rates according to the IMELGA and the INE in the study period for each province. Gray areas indicate a 95% confidence interval.

## Discussion

First, it is necessary to point out, as has been highlighted previously (17), concerns in relation to the evident discrepancy in the data provided by both sources (IMELGA and INE), without either of them being considered more reliable. This indicates a problem in the registration system that must be resolved if we seek to have reliable and accurate data that allows us to know the actual magnitude of the situation and thus be able to intervene effectively. Fortunately, the statistical study reveals that these differences are not relevant and the variation in rates behaves similarly for both databases, as reflected in both the univariate and multivariate studies.

### Differences between provinces

Both the IMELGA and INE data indicate a trend, which remains stable over time with some fluctuations, and which ranks the completed suicide rates from highest to lowest as follows: Lugo > La Coruña > Orense > Pontevedra. This stability in variation is also observed when performing the two-by-two joinpoint regression and observing that parallelism cannot be rejected.

However, accounting for why these differences are maintained over the years is difficult. The variables with the clearest differences between provinces are mean age (Orense > Lugo > La Coruña > Pontevedra) and masculinity ratio (Lugo > Pontevedra > Orense > La Coruña). Both are variables positively related to suicide deaths. In addition, completed suicide is more common in men from rural areas, the most prevalent environment being Lugo and Orense, since these men tend to be stoic and do not seek help from health professionals in the face of mental disorders and stress (18). As these variables are also significant in the LOESS (locally weighted least squares regression) regressions that model the variation in suicide death rates, their relevance is clearly highlighted. Seen together, these two variables provide a robust explanation why Lugo is the province with the most prevalent rates of suicide. However, by themselves they do not enable accounting for the rest of the provinces.

Substance use is another first-order risk factor for suicide death, and the SMR data is a way to measure high risk substance use. SMR is significantly lower in Pontevedra compared to Lugo and La Coruña; and higher compared to Orense. This helps account for Lugo’s position, and sheds light on Pontevedra’s position in bottom place. Even taking this into account, it is difficult to explain the positions of La Coruña and Orense as there are no statistically significant differences between these two provinces and with Lugo.

To account for these unclear results, two causes can be suggested. First, it is possible that there are statistically significant differences between provinces in terms of the prevalence of mental illnesses related to completed suicide, such as depression (19–21). Second, these differences could be related to different styles and interpretation of the procedures in each province when determining cases of suicide deaths, especially in more complex cases. It must be remembered that the prevalence of suicide, although a first-order health problem, is a statistically rare phenomenon; minor variations in the way cases are counted could lead to statistically significant differences between provinces.

### Variation in suicide rates

Joinpoint regression, in its two analyses used in this study, indicates that there is no time of significant change, for both databases, in the variation in suicide rates in each Galician province. It may be difficult to explain the reason for the differences between provinces, but it is clear that these differences remain stable over time.

The most important variables become clear by analyzing the LOESS regression model. First, the masculinity ratio and mean age, variables that cannot be modified by means of preventive interventions. Second, the daily consumption of alcohol and illegal substances. These variables indicate patterns of daily consumption that guide toward dependency and addiction, and therefore to the more serious substance use disorder (SUD). In addition, these variables can be modified by means of prevention and treatment strategies (12). Universal prevention can be useful by means of measures that reduce the overall consumption of alcohol and illegal substances in the general population, thus reducing the incidence of SUDs. In addition, these measures also reduce the risk that alcohol and drugs intoxication becomes a precipitating factor for suicidal behavior in a person who presents suicidal ideation but does not meet the criteria for SUD. Indicated prevention interventions can be suggested to reduce active consumption and its negative consequences in patients presenting SUD and thus minimize suicidal risk. Finally, selective prevention models can be used to undertake suicide prevention plans in patients with SUD at higher risk, due to presenting other associated risk factors such as previous attempts, social maladjustment and psychiatric comorbidity (5, 22, 23).

Currently, the main purpose for the most selective prevention and intervention programmes has been to reduce the risk of retry and completed suicide in patients after an attempted suicide. It is striking that the Intensive Intervention Programme for treating suicidal behavior, which has these same characteristics and has been performed in Orense since 2009, has not proven to be effective (14). In the analysis of the two databases, in no case was there evidence of a positive result when it came to reducing suicide deaths, as reflected in the Joinpoint regression. We formulate two possible explanations for this lack of results: first, this programme has as exclusion criteria for active consumption of alcohol and/or illegal substances, thus excluding patients with a first order risk factor; second, in a 2013 article about this programme, a reduction in new suicide attempts was noted in patients who attend the programme compared to a sample that attends the usual outpatient treatment, However, it is observed that 62.9% of patients included in the programme have a diagnosis of Adjustment Disorder compared to 15.8% of the comparison sample (14). Obviously, Adjustment Disorder presents less severity and less risk. It should be noted that currently there is an important debate about the effectiveness of these programmes, especially about their usefulness as a solo intervention measure. Although they have proven their efficacy, many authors recommend combining them with other interventions (12). It is necessary to remember, despite the statistical results, that these programmes help many patients in an unstable psychopathological situation by means of structured and intensive intervention models.

It is surprising that socio-economic variables, especially unemployment rates, which increased drastically after the economic crisis of 2008/2009, and which persisted until 2015, do not significantly influence the variation in suicide rates. This probably reflects what some authors have already indicated: these are significant variables but with a minor effect (21, 24, 25).

Apart from the problem already mentioned with the discrepancies in the rates of the two databases, this study’s other limitations are the following: the data on substance use and its consequences from the PNSD can be improved. However, it would be desirable to collect more variables related to suicide risk to improve the analysis, especially variables related to severe mental disorder with a particular emphasis on depression.

## Conclusion

Reducing the consumption of alcohol and illegal substances in those people who consume on a daily basis would help reduce the rates of suicide deaths in the Galician provinces. This is because consumption has significantly influenced the variation in the prevalence of suicide in the Galician provinces during the period studied.

## Data availability statement

The original contributions presented in the study are included in the article/supplementary material, further inquiries can be directed to the corresponding author.

## Author contributions

GF, AE, and PS conceptualized the study and designed the methodology. NL and TS-P as biostatisticians assisted in formal statistical analysis. GF wrote the first draft of the manuscript. AE and PS reviewed and edited the manuscript. NL and AE provided input on the analysis and interpretation of the results, and assisted with revising drafts of the manuscript. PS supervised the progress of the study. All authors revised the manuscript critically for important intellectual content and approved the final manuscript.

## Funding

This work has been partially funded by the Government of the Principality of Asturias PCTI-2021-2023 IDI/2021/111, the Foundation for Research and Biosanitary Innovation of the Principality of Asturias (FINBA), and the Centre for Biomedical Research in Mental Health Network (CIBERSAM), Carlos III Health Institute, Spanish Ministry of Science and Innovation.

## Conflict of interest

The authors declare that the research was conducted in the absence of any commercial or financial relationships that could be construed as a potential conflict of interest.

## Publisher’s note

All claims expressed in this article are solely those of the authors and do not necessarily represent those of their affiliated organizations, or those of the publisher, the editors and the reviewers. Any product that may be evaluated in this article, or claim that may be made by its manufacturer, is not guaranteed or endorsed by the publisher.

## Footnotes

^1^https://pnsd.sanidad.gob.es/profesionales/sistemasInformacion/informesEstadisticas/pdf/2021_Summary_report__Informe_OEDA.pdf

^2^https://conselleriadepresidencia.xunta.gal/xustiza/imelga?langId=es_ES

^3^https://www.ine.es

^4^https://pnsd.sanidad.gob.es/profesionales/sistemasInformacion/home.htm

## References

[1] World Health Organization. Estimated suicide worldwide. (2021). Available at: https://www.who.int/mental_health/prevention/suicide/suicideprevent/en/

[2] Knipe D Padmanathan P Newton-Howes G Chan LF Kapur N. Suicide and self-harm. Lancet. (2022) 399:1903–16. doi: 10.1016/S0140-6736(22)00173-8 35512727

[3] Spanish Office for National Statistics (2022). Deaths by cause of death. Year 2021. Definitive data press releases. INE. Available at: http://www.ine.es/prensa/edcm_2021.pdf

[4] Cayuela A Cayuela L Sánchez Gayango A Rodríguez-Domínguez S Pilo Uceda FJ Velasco Quiles AA. Suicide mortality trends in Spain, 1980-2016 [Tendencias de la mortalidad por suicidio en España, 1980-2016]. Rev Psiquiatr Salud Ment. (2020) 13:57–62. doi: 10.1016/j.rpsm.2018.07.002, PMID: 30301678

[5] Wilcox HC Conner KR Caine ED. Association of alcohol and drug use disorders and completed suicide: an empirical review of cohort studies. Drug Alcohol Depend. (2004) 76:S11–9. doi: 10.1016/j.drugalcdep.2004.08.003, PMID: 15555812

[6] Yuodelis-Flores C Ries RK. Addiction and suicide: a review. Am J Addict. (2015) 24:98–104. doi: 10.1111/ajad.12185 25644860

[7] Krug EG Mercy JA Dahlberg LL Zwi AB. The world report on violence and health. Lancet. (2002) 360:1083–8. doi: 10.1016/S0140-6736(02)11133-0 12384003

[8] Beautrais AL Collings S Ehrhardt P Henare K. Suicide prevention: a review of evidence of risk and protective factors, and points of effective intervention. Wellington: Ministry of Health (2005).

[9] Rizk MM Herzog S Dugad S Stanley B. Suicide risk and addiction: the impact of alcohol and opioid use disorders. Curr Addict Rep. (2021) 8:194–207. doi: 10.1007/s40429-021-00361-z, PMID: 33747710 PMC7955902

[10] Kim H Kwon SW Ahn YM Jeon HJ Park S Hong JP. Implementation and outcomes of suicide-prevention strategies by restricting access to lethal suicide methods in Korea. J Public Health Policy. (2019) 40:91–102. doi: 10.1057/s41271-018-0152-x, PMID: 30478435

[11] Zalsman G Hawton K Wasserman D Van Heeringen K Arensman E Sarchiapone M. Evidence-based national suicide prevention taskforce in Europe: a consensus position paper. Eur Neuropsychopharmacol. (2017) 27:418–21. doi: 10.1016/j.euroneuro.2017.01.012, PMID: 28161247

[12] Mann JJ Michel CA Auerbach RP. Improving suicide prevention through evidence-based strategies: a systematic review. Am J Psychiatry. (2021) 178:611–24. doi: 10.1176/appi.ajp.2020.20060864, PMID: 33596680 PMC9092896

[13] Mann JJ Apter A Bertolote J Beautrais A Currier D Haas A. Suicide prevention strategies: a systematic review. JAMA. (2005) 294:2064–74. doi: 10.1001/jama.294.16.2064, PMID: 16249421

[14] Reijas T Ferrer E González A Iglesias F. Evaluation of an intensive intervention program in suicidal behaviour. Actas Espanolas Psiquiatr. (2013) 41:279–86. PMID: 24096393

[15] Llamosas-Falcón L Manthey J Rehm J. Changes in alcohol consumption in Spain between 1990 and 2019. Cambios en el consumo de alcohol en España de 1990 a 2019. Adicciones. (2022) 34:61–72. doi: 10.20882/adicciones.1400, PMID: 32677700

[16] Llanes-Álvarez C Andrés-de Llano JM Álvarez-Navares AI Pastor-Hidalgo MT Roncero C Franco-Martín MA. Trends in psychiatric hospitalization for alcohol and drugs in Castilla y León between 2005 and 2015. Tendencias en la hospitalización psiquiátrica por alcohol y drogas en Castilla y León entre 2005 y 2015. Adicciones. (2022) 34:189–96. doi: 10.20882/adicciones.1405 33338242

[17] Giner L Guija JA. Número de suicidios en España: diferencias entre los datos del Instituto Nacional de Estadística y los aportados por los Institutos de Medicina Legal [Number of suicides in Spain: differences between data from the Spanish Statistical Office and the Institutes of Legal Medicine]. Rev Psiquiatr Salud Ment. (2014) 7:139–46. doi: 10.1016/j.rpsm.2014.01.002, PMID: 24667067

[18] Reccord C Power N Hatfield K Karaivanov Y Mulay S Wilson M. Rural–urban differences in suicide mortality: an observational study in Newfoundland and Labrador, Canada: Différences de la Mortalité par suicide en milieu rural-Urbain: Une Étude Observationnelle à Terre-Neuve et Labrador, Canada. Can J Psychiatr. (2021) 66:918–28. doi: 10.1177/0706743721990315, PMID: 33576277 PMC8573702

[19] Gómez-Durán EL Forti-Buratti MA Gutiérrez-López B Belmonte-Ibáñez A Martin-Fumadó C. Psychiatric disorders in cases of completed suicide in a hospital area in Spain between 2007 and 2010. Rev Psiquiatr Salud Ment. (2016) 9:31–8. doi: 10.1016/j.rpsm.2014.02.001, PMID: 24996402

[20] Onaemo VN Fawehinmi TO D'Arcy C. Risk of suicide ideation in comorbid substance use disorder and major depression. PLoS One. (2022) 17:e0265287. doi: 10.1371/journal.pone.0265287 36477246 PMC9728854

[21] Yoshimasu K Kiyohara C Miyashita K Stress research group of the Japanese society for hygiene. Suicidal risk factors and completed suicide: meta-analyses based on psychological autopsy studies. Environ Health Prev Med. (2008) 13:243–56. doi: 10.1007/s12199-008-0037-x, PMID: 19568911 PMC2698248

[22] Price C Hemmingsson T Lewis G Zammit S Allebeck P. Cannabis and suicide: longitudinal study. Br J Psychiatry. (2009) 195:492–7. doi: 10.1192/bjp.bp.109.065227 19949196

[23] Tiet QQ Ilgen MA Byrnes HF Moos RH. Suicide attempts among substance use disorder patients: an initial step toward a decision tree for suicide management. Alcohol Clin Exp Res. (2006) 30:998–1005. doi: 10.1111/j.1530-0277.2006.00114.x, PMID: 16737458

[24] Iglesias-García C Sáiz PA Burón P Sánchez-Lasheras F Jiménez-Treviño L Fernández-Artamendi S. Suicide, unemployment, and economic recession in Spain. Suicidio, desempleo y recesión económica en España. Rev Psiquiatr Salud Ment. (2017) 10:70–7. doi: 10.1016/j.rpsm.2016.04.005 28238615

[25] Mann JJ Metts AV. The economy and suicide: an interaction of societal and intrapersonal risk factors. Crisis J Crisis Interv Suicide Preven. (2017) 38:141–6. doi: 10.1027/0227-5910/a000487 28641492

